# Associations of CD36 rs1761667 with Fat and Umami Food Perception, Diet Quality, and BMI in Two European Studies

**DOI:** 10.3390/foods14223813

**Published:** 2025-11-07

**Authors:** Francesco Piluso, Catherine Anne-Marie Graham, Harry Stevens, Silvia Camarda, Alexandra King, Leta Pilic, Yiannis Mavrommatis, Paolo Gasparini, Maria Pina Concas

**Affiliations:** 1 Department of Medical Sciences, University of Trieste, 34100 Trieste, Italy; harry.stevens@phd.units.it (H.S.); silvia.camarda@phd.units.it (S.C.); paolo.gasparini@llui.org (P.G.); 2 Lake Lucerne Institute, 6354 Vitznau, Switzerland; cat.maher@llui.org (C.A.-M.G.); yiannis.mavrommatis@llui.org (Y.M.); 3 Faculty of Sport, Technology and Health Sciences, St Mary’s University Twickenham, London TW1 4SX, UK; alexandra.king@stmarys.ac.uk; 4 Optimyse Nutrition Ltd., Radlett WD7 9DJ, UK; leta@optimysenutrition.com; 5 Institute for Maternal and Child Health, IRCCS “Burlo Garofolo”, 34137 Trieste, Italy; mariapina.concas@burlo.trieste.it

**Keywords:** *CD36* gene, fat taste, umami taste, food liking, diet

## Abstract

**Background/Objectives**: Fat, a newly researched taste, has been associated with the cluster of differentiation 36 (*CD36)* gene, which codes for the CD36 receptor protein. The rs1761667 variant has been associated with fat taste sensitivity. Furthermore, umami is a well-established taste with known receptors. The combination of these tastes is common in food; nevertheless, they have not been extensively investigated together. This study aimed to assess whether *CD36* rs1761667 is associated with food liking and the perception of fat and umami foods. **Methods**: Two studies were conducted: A field study on 235 individuals from Italy and a laboratory study on 49 individuals from the UK. Data includes demographics, anthropometrics, a food liking questionnaire or a fat and umami food flavour test, dietary intake assessment, and rs1761667 genotyping. **Results**: Study 1: The rs1761667 A-allele was associated with a reduced liking for fatty and umami foods in individuals with BMI ≥ 25. Study 2: The rs1761667 A-allele was associated with an increased intensity of the umami food samples, and a significant positive association of rs1761667 with BMI and DQS was found. **Conclusions**: This study is the first to address the potential links between rs1761667, fat, umami, diet, and BMI. Further research is required to confirm these findings.

## 1. Introduction

In the past decade, a new taste has been proposed, known as the fat taste or oleogustus [1], due to accumulating evidence of the oral perception of non-esterified fatty acids in humans [2,3]. To date, the health implications of fat consumption, influenced by variations in taste ability, remain unclear. It is known that common genetic variants can influence taste perception [4]. A possible fat-taste receptor is the cluster of differentiation 36 (CD36; codified by the *CD36* gene), a receptor-like glycoprotein expressed in taste bud cells where it binds to long-chain fatty acids [5]. The *CD36* single-nucleotide polymorphism (SNP) rs1761667 (G>A) has been associated with a reduced protein expression [6,7], fat taste hyposensitivity, high dietary fat intake, and an increased risk of developing overweight/obesity [8], although results are inconsistent [9,10,11,12,13]. For example, Melis et al. have observed an association between the *CD36* rs1761667 A-allele and fat taste hyposensitivity [3]. Yet, Graham et al. found no association between *CD36* rs1761667 and fat taste sensitivity [10], and a similar scenario was also reported in Bajit et al., where no association between the AA genotype and oleic acid hyposensitivity was found [9].

Furthermore, carrying the A-allele has been shown to influence the consumption of other tastes in humans [14], and rodent models allow for a hypothesis regarding the interaction of *CD36* with both sweet and umami tastes [15].

Umami is a well-established taste [16]. It is described as savoury and can be perceived in meats, soy sauce, aged cheeses, and other foods [17]. Its main chemical tastant is L-glutamate (glutamic acid). The combination of umami and dietary fat is common in food products (e.g., meats, aged cheeses, etc.).

Both tastes may have negative implications for human health. Individuals with obesity have shown a preference for foods labelled as salty, umami, and fatty [18]. However, the combination of tastes may influence consumption; for example, a preload of monosodium glutamate (MSG), the most common umami compound used for seasoning, can reduce dietary fat intake and salt consumption [19,20,21,22], although clear conclusions cannot be drawn because fat and umami have not been extensively studied together. Therefore, further research is required to assess the perception and liking of fatty foods and umami foods.

To address this gap, this study aimed to investigate whether there is an association between *CD36* rs1761667 and fatty food and umami food liking and flavour perception, habitual dietary intake, and anthropometrics. Two independent studies were carried out. Firstly, study 1, a field survey-based study, investigated whether a relationship exists between rs1761667 and food liking of both fat and umami food products. Secondly, study 2, an exploratory laboratory-based study, investigated whether rs1761667 is associated with liking and intensity ratings of various concentrations of dietary fats in milk and MSG in broth samples. Dietary intake and BMI were assessed in both studies.

## 2. Materials and Methods

### 2.1. Population

The cohort for study 1 (population-based) was a subset from a larger data collection carried out in Friuli-Venezia Giulia (FVG), a region in north-eastern Italy, in 2014–2015, as already described [23]. The study was conducted according to the guidelines of the Declaration of Helsinki and approved by the Ethical Committee and Institutional Review Board of IRCCS “Burlo Garofolo” (under the univocal code Prot. CE/V-78, approval date: 6 August 2007). A total of 235 individuals of European ancestry, as determined by Principal Component Analysis using the genetic data (SNP array), were selected with an age range of 18 to 56 years, both males and females, and with the following data available: BMI, food liking questionnaire, and *CD36* rs1761667 genotype. A subset of dietary intake data was also included.

The cohort for study 2 (laboratory-based) was derived from a novel data collection in the UK. The study was approved by the ethical committee of St Mary’s University (SMU_ETHICS_2021-22_217). Inclusion criteria were any sex, healthy, and aged 18–65 years. Exclusion criteria were pregnancy, being affected by diseases (e.g., hypertension, cardiovascular diseases, or any illness that permanently alters taste, including food allergies), and lactose intolerance. The majority of participants identified themselves as white Europeans (67%). All subjects involved in the studies signed informed consent statements.

### 2.2. Data Collection

Demographic data were collected for both cohorts using a self-administered questionnaire. All questionnaires were conducted on the same day, after a detailed explanation by trained staff. Anthropometric data, including height (m) and weight (kg), were also measured, and body mass index (BMI; kg/m^2^) was calculated.

### 2.3. Genetic Analysis

*CD36* rs1761667 was genotyped in the FVG cohort, as previously described [24]. Briefly, genomic DNA was extracted from blood using a phenol–chloroform extraction procedure, and all samples were genotyped with an Illumina 370/700 k high-density SNP array (Illumina Inc., San Diego, CA, USA), and the genotypes of rs1761667 were extracted.

For the UK cohort, 2 mL saliva samples were provided, and pure genomic DNA was isolated by the PSP^®^ SalivaGene 17 DNA Kit 1011 (Stratec Molecular GmbH, Berlin, Germany), in agreement with the manufacturer’s procedures. Each sample was assessed for quality and quantity using Nanodrop (Thermo Fisher Scientific, Waltham, MA, USA). Genotyping was conducted with a 25 μL reaction volume using the standard thermal cycling parameters and predesigned TaqMan SNP genotyping assay by the quantitative real-time polymerase chain reaction (qPCR) technique (StepOnePlus thermocycler; Applied Biosystems, Foster City, CA, USA). The primers and the probes were pre-designed by Applied Biosystems with the following code: C_8314999_10. The standard thermal cycling parameters were initial denaturation for 10 min at 95 °C, followed by 40 amplification cycles. Each cycle included a 15 s denaturation at 95 °C, followed by one minute annealing/extension at 60 °C. Two negative controls were included during the process. Call rates were all greater than 95%. Two technical replicates of each sample were performed, and individual samples were accepted with a quality of >98%.

### 2.4. Food Liking and Perception

Study 1: Food liking was assessed by questionnaire, due to the absence of a laboratory setting and the numerous of individuals enrolled. The questionnaire investigated liking for more than 100 foods and beverages, using a 9-point hedonic scale: from 1 (dislike extremely) to 9 (like extremely) [25]. Food-liking groups were created, and the Cronbach alpha function available in *R* (package *psy*) was used to assess their reliability. Mean liking value for all foods selected was used for the creation of each group. Non-available data were excluded from the calculation. The names for each group, according to the content, were as follows: Dairy, Cheeses, Other Fatty Foods (meats excluded), Fatty/Umami, and Umami (meats excluded). Single foods used for every group can be found in Supplementary Table S1.

Study 2: Participants were assessed for fat and umami food perception using three food samples per flavour, due to the laboratory setting. Fatty flavour was assessed using commercially available foods with differing fat contents, which is similar to previous the literature [26,27]. Milk samples were selected, specifically, skimmed milk (0.6 g per 200 mL), semi-skimmed milk (3.6 g per 200 mL), and whole milk (7.4 g per 200 mL) (Tesco, Welwyn Garden City, UK). Umami flavour was assessed by dilution of commercially available vegetable stock cubes (OXO, Worksop, UK), prepared according to the manufacturer’s instructions, as per the previous literature [20]. Three MSG (Ajinomoto, Tokyo, Japan) concentrations were used: 0.3%, 0.75%, and 1.5%. Concentrations were piloted internally due to there being no commercially available reference values.

Participants were instructed to swirl the sample in their mouth (~10 s) before expectorating. A five-minute break was given between the fatty and umami tastings. A visual analogue scale (VAS) (0.0 cm = lowest to 10.0 cm = highest) was used to rate intensity and liking perceived, as per the previous literature [28].

### 2.5. Dietary Intake and Diet Quality Score (DQS)

Study 1: Dietary intake data were available only in the 48 subjects who agreed to attend a dietary history interview, conducted by professionals, as previously described [29]. The interview evaluated the average daily food intake for each participant. Nutrient intake was then assessed through software (WinFood, 2.7. Medimatica, San Benedetto del Tronto, Italy) based on Italian food composition tables.

Study 2: Dietary intake data were measured using two 24 h 5-step multiple pass recalls. Participants provided information regarding all food and drinks consumed within the past 24 h period, together with quantities and a detailed description. The recall was administered online using the JISC survey platform (Join Information Systems Committee, London, UK).

In both studies, dietary intake data were used to assess compliance with the dietary guidelines in each respective country [30,31], creating a DQS. Each dietary component was rated from 0 for non-compliance to 1 for compliance. In study 1 the following data were available: carbohydrates (g/die), total fats (g/die), saturated fatty acids (g/die), fibre (g/die), and sodium (mg/die), while in Study 2 free sugars (g/die), total fats (g/die), saturated fatty acids (g/die), fibre (g/die), and sodium (mg/die) were available. The final DQS assigned to each participant ranged from 0 (no compliance at all) to 5 (compliance for every component).

### 2.6. Statistical Analyses

In study 1, the effect of the *CD36* rs1761667 genotypes on food liking groups, DQS, and BMI was assessed using linear regression models. An additive inheritance model was applied to all the analyses performed (0 = GG, 1 = GA, 2 = AA). Then, the possible association between food liking groups and DQS was investigated using the linear regression model.

In study 2, the differences in intensity and liking ratings for the three fat and umami samples were evaluated using repeated measures analysis of variance (ANOVA), with fat and MSG concentration as the repeated measures for the respective food samples (within factor). The main effect of *CD36* rs1761667 genotypes (between factor) as well as the interactions with concentrations were examined in the ANOVA models. Testing for normality was performed with the Shapiro–Wilk test. Post hoc comparisons were made using Tukey’s test following Bonferroni adjustment. The effect of *CD36* rs1761667 genotypes on DQS and BMI was investigated using the same statistical approach as in study 1. Then, the relationship between the perception of the fatty and umami food samples was assessed by Pearson correlation, and as an exploratory analysis, we assessed the possible association between fatty and umami food perception on DQS by a linear regression model.

Age, sex, and BMI were included in the model as covariates for every analysis performed. In study 1, participants were divided into those with a BMI ≥ 25 kg/m^2^ (overweight and with obesity) and those with a BMI < 25 kg/m^2^ (healthy weight and underweight) according to the WHO guidelines in adults [32]. Stratification by sex was also assessed.

R studio was used throughout (R version 4.4.0 (2024-04-24 ucrt) -- “Puppy Cup” Copyright (C) 2024, The R Foundation for Statistical Computing). Throughout, the significance criterion was *p* ≤ 0.05. The Hardy–Weinberg equilibrium (HWE) of *CD36* rs1761667 was assessed in both studies using the “R” package “genetics”.

We performed a priori power analysis using G*Power (version 3.1 for Mac) [33,34]. As there was limited prior research, we assumed a medium effect size of the SNP for both studies. For study 1, which employed a linear multiple regression with four predictors, a sample of 55 individuals was necessary to detect a medium effect size (f^2^ = 0.15) for a single predictor with 80% power at alpha = 0.05. Regarding study 2, for which a mixed ANOVA design was used (between-subjects: 3 groups; within-subjects: 3 measurements), a sample of 36 participants (12 per group) was needed to detect a medium effect size (Cohen’s f = 0.25) with 80% power at alpha = 0.05.

## 3. Results

### 3.1. Study 1: FVG

#### 3.1.1. Participants’ Characteristics and Food Liking

Two hundred and thirty-five participants were analysed, as summarised in Table 1.

Cronbach’s alpha value was >0.5. The *CD36* rs1761667 genotype followed HWE (*p* > 0.05).

#### 3.1.2. Associations Between CD36 rs1761667 and Food Liking

A summary of all linear regression models is reported in Table 2. No significant association was found between CD36 rs1761667 and liking for Dairy, Cheeses, Other Fatty Food, Umami/Fatty Meats, or Umami (No Meat) groups (*p* > 0.05, Table 2). Models revealed that BMI was a positive significant predictor for the liking of Cheeses (β = 0.04, SE = 0.02, *p* = 0.05), Other Fatty Foods (β = 0.07, SE = 0.02, *p* = 0.03) and for, in male participants only, for Fatty/Umami group liking (β = 0.05, SE = 0.03, *p* = 0.05) (Supplementary Table S3).

When stratified by BMI, in those with a BMI ≥ 25 kg/m^2^, a significant, negative association was found between the rs1761667 A-allele (β = −0.47, SE = 0.18, *p* = 0.01) and liking Cheeses and with liking Umami (no meat) (β = −0.47, SE = 0.16, *p* = 0.02). No other statistically significant associations were found with BMI, nor with sex.

#### 3.1.3. Associations Between CD36 rs1761667 and DQS, and DQS and Food Liking

Regarding the DQS, no significant associations were found (Supplementary Table S2). No stratification was applied due to the low number of individuals with available dietary data.

#### 3.1.4. Associations Between CD36 rs1761667 and BMI

No significant association was found between *CD36* rs1761667 and BMI, even after stratification according to BMI (≥/<25 kg/m^2^) and sex (*p* > 0.05).

### 3.2. Study 2: UK

#### 3.2.1. Participants’ Characteristics and Food Perception

A total of 49 participants were analysed, as summarised in Table 3. The *CD36* rs1761667 genotype followed HWE (*p* > 0.05).

#### 3.2.2. Associations Between CD36 rs1761667 and Food Sample Perception

**Figure 1:**
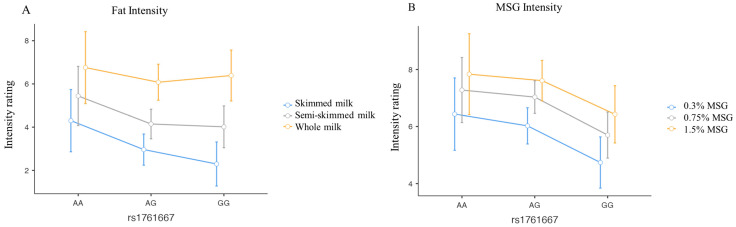
Line plots describing the perceived intensity of fat (**A**) and umami (**B**) flavour in food samples for each concentration, according to *CD36* rs1761667 genotype. Blue lines represent the lowest concentration, grey solid lines represent the intermediate concentrations, and yellow lines represent the highest concentration. The legend is reported on the right for both graphs. Dots represent the average value for every genotype, and bars represent the standard error. The effect of different concentrations is statistically significant for both fatty and umami samples (*p* < 0.001), while the effect of the SNP is significant only for umami (*p* = 0.009), for which the GG carriers perceived the lowest intensity compared to AA and AG individuals at each concentration. No interaction effect was detected (*p* = 0.963).

A statistically significant effect of the concentration of fat on the perceived intensities from the three milk samples was found (*p <* 0.001). More specifically, post hoc tests revealed that each comparison between the intensities reported for the three different concentrations (skimmed, semi-skimmed, and whole) was statistically significant (*p* < 0.05). Nevertheless, we found no significant effect of the *CD36* rs1761667 genotype (*p* = 0.252) (Figure 1A).

Regarding the umami perception, both the effect of the three different MSG concentrations (*p* < 0.001) and the *CD36* rs1761667 genotype were detected (*p* = 0.009) (Figure 1B). No interaction between concentration and genotypes was observed (*p* = 0.9). All comparisons of intensity between different concentrations (0.3%; 0.75%; 1.5%) were statistically significant, as were the comparisons between genotypes, except for the comparison between AA and AG.

Additionally, we found no statistically significant effect of concentrations or *CD36* rs1761667 genotype, for both fatty and umami liking in food samples (*p* > 0.05).

#### 3.2.3. Correlation Between Intensity and Liking for Fat and Umami Food Samples

**Figure 2:**
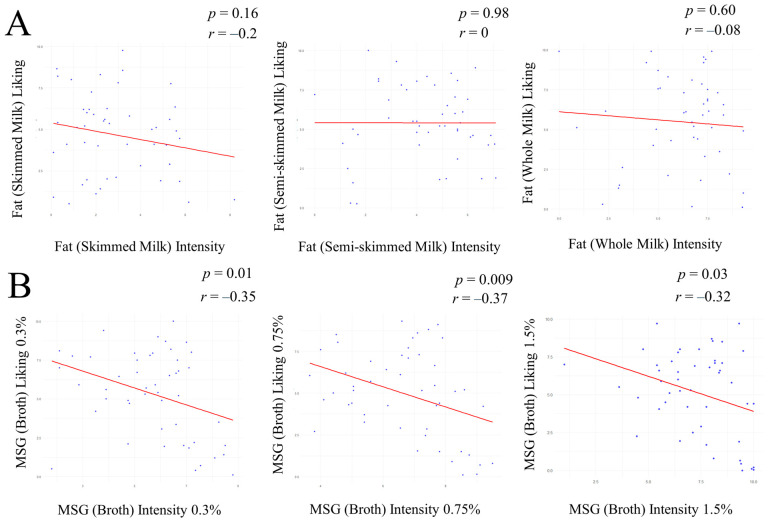
Scatter plot illustrating the correlation between intensity and liking for the three fat (milk) samples (**A**) and for the three MSG (broth) samples (**B**). The X-axis represents intensities for each sample. The Y-axis represents liking for each sample. Points represent individual observations, while the solid red line indicates the linear regression line and direction of the correlation. The Pearson correlation coefficient (*r*) and *p*-value (*p*) are indicated, for every analysis, in the top right corner of each plot.

A significant, negative correlation was observed between the intensity and liking of umami food samples (Broth 0.3% MSG, cor = −0.35, *p* =0.01; Broth 0.75% MSG, cor = −0.37, *p* = 0.009; Broth 1.5% MSG, cor = −0.32, *p* = 0.03; Figure 2B). No correlation was observed between the intensity and liking of fatty food samples (Figure 2A), or the intensity of fatty and umami food samples, the liking of fatty and umami food samples, the intensity of fatty food samples and the liking of umami food samples, and the intensity of umami food samples and the liking of fatty food samples (*p* > 0.05; Supplementary Table S4).

#### 3.2.4. The Role of CD36 rs1761667 on DQS and BMI

A significant, positive association was found between the *CD36* rs1761667 A-allele and DQS (*β* = 0.64, *SE* = 0.30, and *p* = 0.03). Additionally, the *CD36* rs1761667 A-allele was associated with an increased BMI (*β* = 2.0, *SE* = 0.8, *p* = 0.02). However, BMI as a covariate showed no significant association with DQS.

#### 3.2.5. The Role of Food Sample Perception on DQS

As an exploratory analysis, the possible association between fat and umami perception with DQS and BMI was investigated. A significant, positive association was found between intensity perceived from the skimmed milk sample and DQS (*β* = 0.2, *SE* = 0.13, *p* = 0.04). Additionally, a significant, negative association was found between liking of the whole milk sample and DQS (*β* = −0.14, *SE* = 0.06, *p* = 0.03). No other associations regarding DQS were apparent (*p* > 0.05) (Supplementary Table S5).

## 4. Discussion

In this work, the *CD36* rs1761667 SNP was investigated in two independent studies to contribute to a better understanding of its role in fat and, potentially, umami food perception.

### 4.1. CD36 rs1761667, Food Liking, BMI, and DQS in Study 1

In the population-based study, for which food liking was assessed by questionnaire, the *CD36* rs1761667 A-allele was negatively associated with liking of the fatty food group “Cheeses” in participants with a BMI ≥ 25 kg/m^2^. This result contrasts with Keller et al., where AA genotype carriers reported a higher liking for the “added fats and oil” food group, and this was also assessed by questionnaire [35], and Shen et al., where no significant differences in the liking of ice cream with differing fat contents were found, assessed by food tasting [36]. Additionally, in study 1, the A-allele was associated with reduced liking of the umami food group in participants with a BMI ≥ 25 kg/m^2^. Dietary fats are present in umami-based foods; for example, in hard cheeses, fat content is generally >30g/per serving, and in sardines, fat content is approximately 5 g/per serving [37]. This may account for the genetic association between *CD36*, the possible fat taste receptor gene, and the liking of umami foods, which highlights the complex interplay between flavours and food liking. Our study is not the first to identify an association between genetics and umami food liking in an SNP that has previously been associated with a different taste. Han et al. reported an association between *TAS1R3* rs307355 and rs35744813 and a higher consumption of protein-rich and savoury foods in a buffet setting [38,39].

Furthermore, these results support the concept that BMI is associated with fatty and umami food liking, as observed in previous works [40], and that this may alter food choices [41,42,43,44,45]. Nevertheless, the effect size reported between BMI and food-liking groups, even if statistically significant, is minimal, and no final conclusions can be reached regarding health outcomes. It has been proposed that obesity may encompass gene expression in type II taste bud cells [46], which may be involved in free fatty acid stimulus perception [47], possibly causing food-liking variability across the general population. This warrants further research assessing genetic differences in taste receptors and food liking, whilst considering body composition.

Whether taste receptor genes that have been associated with food liking influence actual dietary intake is not known. In study 1, there was no association with rs1761667 and dietary intake. Whether taste receptor genes that have been associated with food-liking influence actual dietary intake is not known, with the previous literature reporting contrasting results [48,49,50]. Lifelong habitual dietary intake, largely determined by culture, may impact food liking. This may influence results related to Italy due to the “Mediterranean” dietary pattern [51], which is high in fats, being a strong cultural behaviour. Further research using a comprehensive dietary assessment is required to confirm this.

### 4.2. CD36 rs1761667 and Food Sample Perception in Study 2

In this exploratory laboratory-based study, the role of genetics and food perception was assessed.

Regarding fat, no associations were found between the *CD36* rs1761667 SNP and intensity of perception or liking. In contrast, Melis et al. found that carriers of the AA genotype showed reduced sensitivity to oleic acid compared to carriers of the GG genotype [3]. Nevertheless, multiple studies have failed to demonstrate any associations, as seen in our work [10,52]. The heterogeneity reported in the literature may be due to the different methodologies adopted to investigate fat taste; for example, this study (study 2) utilised commercially available foods, as per the previous literature [26,27]. While this allows for better translation to nutrition practice, whole foods are often a complex mixture of tastes with a vast array of fat types and concentrations that may confound results. However, even with consistent, simple taste mediums, results can potentially differ due to other sensory aspects, i.e., texture, which can also influence results when using whole foods. For example, Daoudi et al. utilised oleic acid water emulsion to evaluate fat-taste sensitivity [52], finding no association with rs1761667, contrasting with the association reported in Graham et al., which used oleic acid fat-free UHT milk emulsion [53], considered to be more homogeneous than water emulsion [54]. Further research is required to assess the differences between fat-taste assessment vehicles and their relationship to fat-taste receptor genetic variability to ensure clear conclusions can be drawn.

Regarding umami, in this study, there was an association between rs1761667 and umami flavour perception but not liking. This is the first time, to our knowledge, that an association between umami flavour perception and *CD36* has been reported. However, associations between other genetic variants and umami perception have previously been shown (e.g., *TAS1R1* rs11122100 and rs12080675) [4], and genetic interactions between taste perception and dietary intake have been reported; for example, the *TAS1R1* rs34160967 SNP has been associated with umami taste perception [55] and is separately related to a higher dietary fat intake [38]. Additionally, the *TAS1R1* rs34160967 SNP has been associated with umami, sweet, and salty taste sensitivity [56].

All considered, and accounting for the exploratory nature of our study, our results warrant further research to assess the genetic interactions between all known taste-related genes and all known tastes, and whether this may influence dietary intake and other health markers.

### 4.3. Correlation Between Intensity and Liking for Food Samples in Study 2

Study 2 demonstrates a negative correlation between intensity and liking for all the umami food samples, following the assumption that the liking ratings vary according to the sensation intensity [57]; however, this was not reported for the fatty food samples. The correlation between intensity and liking may change depending on the taste studied [58]. Additionally, the familiarity of foods assessed may be confounding; for example, individuals may show a higher liking for the type of milk they have regularly been exposed to [59]. Future research should be carried out to further validate the results previously discussed, including all known tastes, with an adequate sample size, which will provide better insight into how we perceive tastes and how this can influence our food choices.

### 4.4. CD36 rs1761667 and DQS, and DQS and Food Perception in Study 2

Taste perception has previously been identified as a driver for different dietary patterns [60], as well as genetics [48]. Study 2 revealed a positive association with DQS and the *CD36* rs1761667 A-allele and with skimmed milk sample intensity. Conversely, liking for the whole milk sample was negatively associated with DQS. The role of *CD36* rs1761667 on dietary intake has been investigated previously. For example, Fujii et al., in a Japanese cohort, observed a positive association between total fat intake and the *CD36* rs1761667 A-allele. In contrast, Pioltine et al. found the same allele associated with a lower daily fat consumption in a South American cohort [49], while Chmurzynska et al. reported, in a Polish cohort, no association with fat food consumption [50]. These works investigated three different populations and used three different methodologies to assess dietary intake (food frequency questionnaire, 24 h food recalls, and a mobile phone application, respectively), making direct comparison challenging. Dietary intake data is commonly considered limited and challenging to interpret [61], which may explain the heterogeneity in observed results. Taste is one of the major factors that influences dietary choice [62,63,64], and thus the potential effect that *CD36* rs1761667 may have on food perception, and the potential effect of food perception on dietary intake and quality, warrants further research.

### 4.5. CD36 rs1761667 and BMI in Study 2

Fat food consumption has previously been associated with increased adiposity parameters [9,65]. Here, we report that rs1761667 A-allele carriers had an increased BMI, which is a disease risk indicator [66] that has been linked to eating habits [67]. However, BMI, although associated with body fat, does not directly measure body fat [68]. When discussing body fat and taste perception, various mechanisms have been hypothesised related to increasing dietary intake [69], including reward circuitry and microbiota, factors that are related to body composition, of which BMI does not reliably measure. Therefore, to better understand the relationship between rs1761667 and adiposity alluded to in this current study, it is recommended that future research integrate measurements of body composition (hip-to-waist ratio/bio-electrical impedance/dual-energy X-ray absorptiometry) with an adequate sample size for such stratification.

## 5. Conclusions

In our studies, results show that the A-allele is negatively associated with food liking in participants who are overweight or obese and is positively associated with the perceived intensity of umami foods. Moreover, a positive relationship between the A-allele and BMI and elements of diet quality was found. Nevertheless, additional research activities on larger cohorts should be carried out to confirm the present findings.

Furthermore, considering that whole foods’ gustatory features cannot be associated solely with one taste, future research should explore the interactions between all six researched tastes, including their genetic receptor variability. This manuscript is the first to investigate the potential links between the *CD36* gene, flavour perception, the liking of fat and umami foods, and food liking on a population level, with comments on dietary patterns and BMI implications. While these preliminary findings require further validation in larger and better characterised cohorts, due to the limitations previously highlighted, they represent a starting point for translating scientific observations into the real world.

## Figures and Tables

**Table 1:** Participants’ features and food-liking scores described according to the *CD36* rs1761667 genotype in study 1 (FVG cohort).

	Total (n = 235)	AA rs1761667 (n = 58, 25%)	AG rs1761667 (n = 108, 46%)	GG rs1761667 (n = 69, 29%)
Female % (n)	54 (128)	45 (26)	56 (61)	59 (41)
Age (years), mean ± SD	34 ± 9.2	33 ± 9.7	34 ± 9.3	34 ± 8.8
BMI (kg/m^2^), mean ± SD	24.0 ± 4.4	24.0 ± 5.1	24.0 ± 4.2	24.0 ± 4.2
BMI ≥ 25 (kg/m^2^), mean ± SD	28.8 ± 3.8	29.2 ± 5.2	28.5 ± 3.3	28.7 ± 2.9
BMI < 25 (kg/m^2^), mean ± SD	21.6 ± 2.2	21.7 ± 2.4	21.6 ± 2.2	21.6 ± 2.1
Dairy, mean ± SD	6.0 ± 1.2	6.1 ± 1.2	5.8 ± 1.1	6.2 ± 1.3
Cheeses, mean ± SD	6.3 ± 1.4	6.4 ± 1.5	6.1 ± 1.4	6.6 ± 1.5
Other Fatty Foods, mean ± SD	6.2 ± 1.5	6.0 ± 1.7	6.2 ± 1.5	6.3 ± 1.4
Fatty / Umami, mean ± SD	6.3 ± 1.4	6.3 ± 1.4	6.4 ± 1.4	6.2 ± 1.6
Umami foods (meats excluded), mean ± SD	5.9 ± 1.2	5.9 ± 1.1	5.7 ± 1.3	6.3 ± 1.2
DQS, mean ± SD *	2.4 ± 1.3	2.7 ± 1.5	2.6 ± 1.2	1.9 ± 1.3

FVG, Friuli-Venezia Giulia; AA/AG/GG, rs1761667 genotype; DQS, diet quality score; SD, standard deviation; n: sample size. * DQS data available for 48 participants.

**Table 2:** Results of the linear regression models testing the association between the SNP rs1761667 (independent variable) and food liking (dependent variable) in all samples and stratified by BMI and sex.

	All FVG (n = 235)		BMI < 25 kg/m^2^ (n = 155)		BMI ≥ 25 kg/m^2^ (n = 80)		Only Females (n = 128)		Only Males (n = 107)	
Food Groups	*β (SE)*	*p*	*β (SE)*	*p*	*β (SE)*	*p*	*β (SE)*	*p*	*β (SE)*	*p*
Dairy	−0.07 (0.10)	0.5	0.08 (0.10)	0.5	−0.33 (0.20)	0.06	−0.08 (0.15)	0.6	−0.06 (0.15)	0.7
Cheeses	−0.14 (0.13)	0.3	0.05 (0.17)	0.7	−0.47 (0.18)	0.01 *	−0.20 (0.19)	0.3	−0.07 (0.17)	0.7
Other Fatty Foods	−0.19 (0.13)	0.1	−0.17 (0.17)	0.3	−0.18 (0.19)	0.3	−0.08 (0.19)	0.7	−0.29 (0.17)	0.09
Fatty/Umami	0.002 (0.12)	0.9	0.04 (0.15)	0.8	−0.07 (0.21)	0.8	−0.01 (0.20)	0.9	0.008 (0.14)	0.9
Umami foods	−0.18 (0.11)	0.1	−0.03 (0.13)	0.8	−0.47 (0.16)	0.02 *	−0.23 (0.16)	0.2	−0.23 (0.16)	0.2

β, effect size (beta value); SE, standard error; *p*, *p*-value; the following covariates were included in the models: age, sex, and BMI, in the whole sample; age and sex in the BMI subsamples; and age and BMI in the sex specific analyses. * Indicates significant results.

**Table 3:** Participants’ features and food sample perception and liking according to *CD36* rs1761667 genotype in study 2 (UK cohort).

		Total (n = 49)	AA rs1761667 (n = 7, 14%)	AG rs1761667 (n = 28, 57%)	GG rs1761667 (n = 14, 29%)
Female % (n)		63 (31)	86 (6)	57 (16)	64 (9)
Age (year), mean ± SD		32 ± 11.1	36 ± 12.3	30 ± 9.7	35 ± 12.8
BMI (kg/m^2^), mean ± SD		23.6 ± 3.9	24.7 ± 2.5	24.4 ± 4.4	21.4 ± 2.6
Fat (Milk) Intensity	Skimmed	2.9 ± 1.9	4.3 ± 1.7	2.9 ± 2.2	2.3 ± 1.3
	Semi-skimmed	4.3 ± 1.8	5.4 ± 1.5	4.2 ± 1.8	4.0 ± 1.9
	Whole	6.3 ± 2.2	6.8 ± 1.8	6.1 ± 2.1	6.4 ± 2.5
Fat (Milk) Liking	Skimmed	4.6 ± 2.4	5.2 ± 2.2	4.1 ± 2.4	5.4 ± 2.4
	Semi-skimmed	5.4 ± 2.4	5.8 ± 2.4	5.0 ± 2.6	5.9 ± 1.9
	Whole	5.5 ± 2.9	5.2 ± 3	5.0 ± 2.9	6.5 ± 2.8
MSG (Broth) Intensity	0.3%	5.7 ± 1.8	6.4 ± 1.1	6.0 ± 1.7	4.7 ± 1.9
	0.75%	6.7 ± 1.6	7.3 ± 1.4	7.0 ± 1.5	5.7 ± 1.6
	1.5%	7.3 ± 1.9	7.8 ± 1.0	7.6 ± 2.1	6.4 ± 1.5
MSG (Broth) Liking	0.3%	5.3 ± 2.7	6.0 ± 2.5	4.9 ± 2.9	5.8 ± 2.3
	0.75%	4.9 ± 2.6	5.9 ± 2.6	4.4 ± 2.7	5.7 ± 2.3
	1.5%	5.1 ± 2.8	5.8 ± 2.7	4.8 ± 2.9	5.5 ± 2.6
Diet Quality Score, mean ± SD		1.8 ± 1.3	2.6 ± 1.4	1.8 ± 1.2	1.5 ± 1.2

UK, United Kingdom; AA/AG/GG, rs1761667 genotype; SD, standard deviation; n, sample size; MSG, monosodium glutamate; DQS: diet quality score. In the second column, the type of milk (based on the concentration of fat) is reported, while for the broth, the concentration of MSG is reported.

## Data Availability

A subset of the data is already available in the European Genome-phenome Archive (EGA) at the following links. FVG cohort: BAM files https://www.ebi.ac.uk/ega/studies/EGAS00001000252 (accessed on 13 November 2023); sample list, vcf files https://www.ebi.ac.uk/ega/studies/EGAS00001001597 (accessed on 13 November 2023); https://www.ebi.ac.uk/ega/datasets/EGAD00001002729 (accessed on 3 November 2023). The raw data supporting the conclusions of this article from the United Kingdom will be made available by the authors on reasonable request.

## Supplementary Materials

The following supporting information can be downloaded at https://www.mdpi.com/article/10.3390/foods14223813/s1; Table S1: FVG_foodgroup, Table S2: FVG_DQS_models, Table S3: FVG_BMI_models, Table S4: UK_cor.test, and Table S5: UK_DQS_perception.

## Abbreviations

The following abbreviations are used in this manuscript:

CD36	Cluster of differentiation 36
SNP	Single-nucleotide polymorphism
MSG	Monosodium glutamate
FVG	Friuli Venezia Giulia
UK	United Kingdom
BMI	Body mass index
NA	Non-available data
VAS	Visual analogue scale
DQS	Diet quality score
HWE	Hardy–Weinberg equilibrium
SD	Standard deviation
SE	Standard error
